# BCG Scar Local Skin Inflammation as a Novel Reaction Following mRNA COVID-19 Vaccines in Two International Healthcare Workers

**DOI:** 10.7759/cureus.14453

**Published:** 2021-04-13

**Authors:** Erika Z Lopatynsky-Reyes, Heidy Acosta-Lazo, Rolando Ulloa-Gutierrez, María L Ávila-Aguero, Enrique Chacon-Cruz

**Affiliations:** 1 Family Medicine and Public Health, University of California San Diego, San Diego, USA; 2 Servicio de Infectología, Hospital Nacional de Niños "Dr. Carlos Sáenz Herrera" Centro de Ciencias Médicas, Caja Costarricense del Seguro Social (CCSS), San José, CRI; 3 Cátedra de Pediatría, Universidad de Ciencias Médicas (UCIMED), San José, CRI; 4 Pediatric Infectious Diseases, Center for Infectious Disease Modeling and Analysis (CIDMA) Yale School of Public Health, New Haven, USA; 5 Departamento de Infectología, Hospital General de Tijuana, Tijuana, MEX

**Keywords:** bcg, bcg scar reaction, covid 19, sars-cov-2 vaccines, sars-cov-2 vaccine reactogenicity, vaccine adverse reactions, vaccine adverse events

## Abstract

Bacillus Calmette-Guérin (BCG) local scar inflammatory reactions have been mostly associated with Kawasaki disease in children and less commonly with other viral infections (i.e., measles). BCG scar inflammation associated with or following vaccine administration has only been reported with the influenza vaccine. We describe the first reports in the literature of local BCG inflammation following two different available messenger ribonucleic acid (mRNA) anti-severe acute respiratory syndrome coronavirus 2 (anti-SARS-CoV-2) vaccines (mRNA-1273, and BNT162b2) in two young healthy physicians, one from Costa Rica, and another from the United States of America, with normal cell blood counts, flow cytometries, and negative for human immunodeficiency virus (HIV). In both cases, BCG scar inflammation appeared after 24 hours of vaccination of the second dose, without signs of reaction on the injection site, and resolved within four days. Dermoscopic findings in one case showed arborizing and comma-shaped vessels. Pharmacovigilance surveillance of BCG scar reactions following coronavirus disease 2019 (COVID-19) vaccines should be considered particularly in countries where BCG is part of their national immunization programs.

## Introduction

Inflammatory reactions of the Bacillus Calmette-Guérin (BCG) scar have been mostly reported as a feature in children with Kawasaki disease (KD), particularly in the first two years of life, as it has previously been described [1-5]. This phenomenon is characterized by erythema, induration, ulceration, and/or crust formation at the inoculation site. Nonetheless, it has also been described with measles and human herpesvirus type 6 (HHV6) infection [6-7].

To our knowledge, BCG scar inflammation following immunization has only been published twice in two children from Japan and Mexico, respectively, following influenza vaccination [8-9].

In this report, we describe a novel finding of two cases in healthcare workers from Costa Rica and the United States of America (USA), respectively, of a BCG scar inflammatory reaction following the second dose administration of the two currently available messenger ribonucleic acid (mRNA) coronavirus disease 2019 (COVID-19) vaccines (BNT162b2 and mRNA-1273).

## Case presentation

Following the administration of the second dose of the currently available mRNA COVID-19 vaccines, two previously healthy healthcare workers from two different countries (Costa Rica and the USA) reported having a local-adjacent reaction characterized by erythema and induration of the BCG scar, 3-5 cm from the vaccine injection site.

Case 1

On December 30, 2020, a previously healthy, 31-year-old female pediatrics resident living in San Jose, Costa Rica, who was vaccinated with BCG at birth on her right shoulder, with an irrelevant past medical history, received the first dose of mRNA (BNT162b2, Pfizer®, New York, New York) SARS-CoV-2 vaccine on her left arm, and it was not associated with any adverse reactions. Twenty-one days later, determined by the scheduled appointment to receive the second dose in Costa Rica, received her second vaccine dose on the right arm without presenting any immediate side effects. Twelve hours later, the patient had headaches, chills, and myalgias in the upper and lower limbs. The following day, she also complained of pain at the site of the injection and on the ipsilateral trapezius muscle. No fever was detected. Three doses of oral paracetamol were given with partial improvement of the symptoms. Throughout the day, trapezius muscle pain increased and two lymphadenopathies were found within the muscle, about 0.5 cm each, mobile, and painful to palpation.

**Figure 1:**
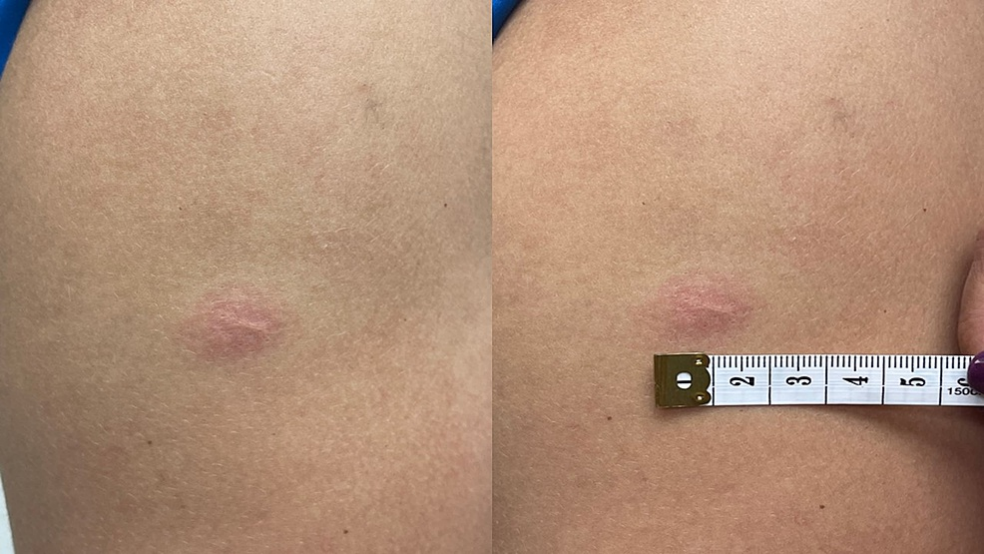
Case 1 - BCG scar inflammation 5 cm below injection (vaccine) site BCG: Bacillus Calmette-Guérin

On the second day post-vaccine, an inflammation area of 1.5 cm on the BCG scar site was noted with erythema, induration, but painless to palpation, five cm from the injection (vaccination) site (Figure 1). No other symptoms were present on that day. The inflammation on the BCG scar lasted four days and the lymphadenopathies on the right trapezius muscle resolved after eight days following the vaccine application. Complementary laboratories such as human immunodeficiency virus (HIV)-enzyme-linked immunosorbent assay (ELISA), cell blood count (CBC), and flow cytometry were performed to discard any potential immunodeficiencies. All results were normal (Table 1).

**Table 1:** CBC, flow cytometry, and HIV-ELISA from both cases CBC: Cell blood count; HIV: Human immunodeficiency virus; ELISA: Enzyme-linked immunosorbent assay; WBC: White blood cells

Laboratory results			
	Normal Values	Case 1	Case 2
Hemoglobin	12-16	12.1	13.3
Platelets	140,000 - 440,000	295,000	225,000
WBC – total	4,500 – 11,000	5,080	7,900
Neutrophils – total	1,800 – 7,700	2,550	5,846
Lymphocytes - total	1,000 - 4,800	1,828	1,422
Lymphocytes T - CD3 (%)	56.5 -84.7%	58%	70.66 %
Lymphocytes T – CD4 (%)	30.3-58%	36%	44.56 %
Lymphocytes T – CD8 (%)	17-33%	21%	24.64 %
HIV-ELISA	Negative	Negative	Negative

Case 2

This is a healthy, with no clinically relevant medical history, 28-year-old female medical doctor born in Tijuana, Mexico (received BCG immunization at birth on her right shoulder) but with USA citizenship and currently working in San Diego, California, USA. The first dose of mRNA-1273 COVID-19 vaccine (Moderna®, Moderna, Inc, Cambridge, Massachusetts) vaccine was administered on January 17, 2021, on the left arm; the patient referred pain, redness at the injection site, myalgias, arthralgias, and malaise, lasting only two days.

The second dose was given on the right arm on February 23, 2021 (36 days apart due to vaccine supply shortage in the region and re-scheduling of the appointment). Following the administration of the second dose, she also complained of headache, nausea, myalgias, arthralgias, and malaise lasting for two days only, and treated with four doses of paracetamol.

**Figure 2:**
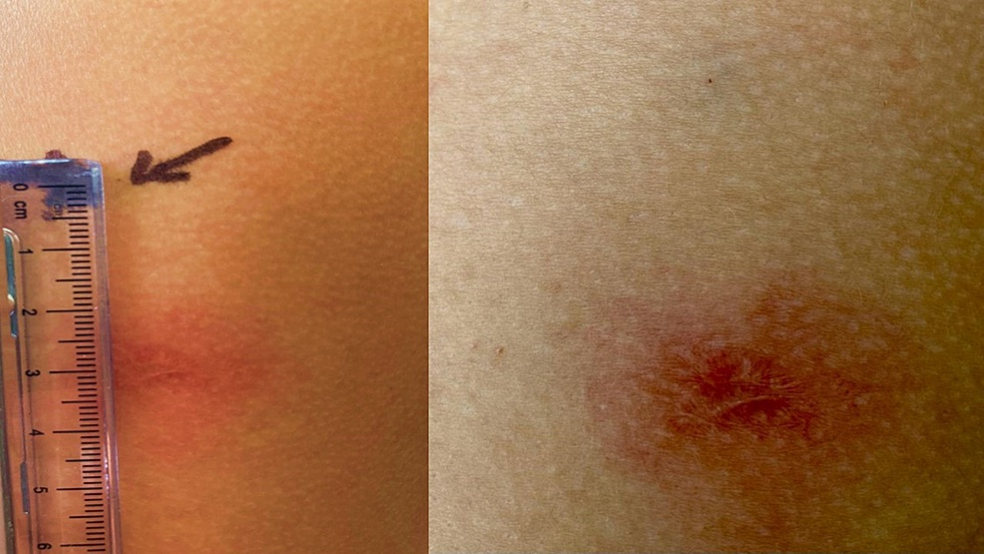
Case 2 - BCG scar inflammation 3 cm below injection (vaccine) site BCG: Bacillus Calmette-Guérin

In addition, she presented an erythematous reaction followed by pain, induration, and mild edema at her BCG scar site (Figure 2) 36 hours after receiving the second dose. Complementary laboratories, such as HIV-ELISA, CBC, and flow cytometry, were performed to discard any potential immunodeficiencies in both cases. All results were normal (Table 1).

**Figure 3:**
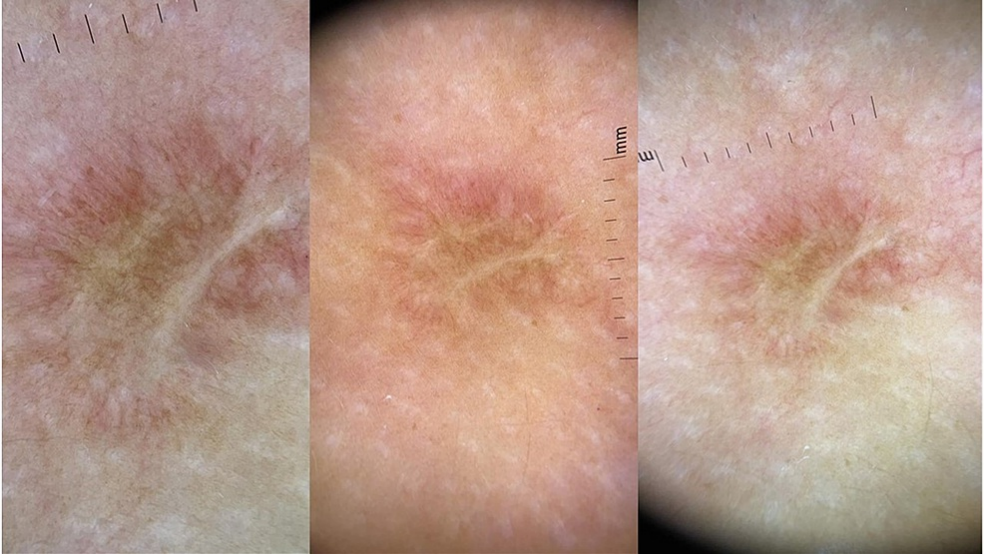
Case 2 - BCG scar dermoscopic findings of inflammation and presence of "arborizing and comma-shaped" vessels BCG: Bacillus Calmette-Guérin

Furthermore, a dermatological evaluation was performed by a dermatologist, describing a dermatosis affecting the proximal right upper extremity with an erythematous BCG scar of 10 x 12 mm, three centimeters from the injection site; moreover, a dermoscopy (by using a Dermlite® II Pro polarized light dermatoscope; 3Gen Inc., San Juan Capistrano, CA) was performed on the BCG scar, identifying a hypochromic stellar pattern with "arborizing" and "comma-shaped" vessels (Figure 3). The reaction lasted for two days with no further affection.

## Discussion

Although not specific, the dermatological changes at the BCG inoculation site scar have been also described in KD, particularly in young infants, even though its mechanism is not fully understood. However, in children older than two years of age and, even more, in adolescents or adults suffering from KD, this reaction is extremely rare [1-5]. Other case reports have been published in association with viral infections such as measles and HHV6 [6-7]. However, this inflammatory response following immunization has only been published twice. These reports are from a nine-year-old Mexican child and a two-year-old Japanese infant, who two days after receiving influenza vaccination developed pain, erythema, and tenderness at the BCG scar [8-9]. To our knowledge, BCG reactivation as a local-adjacent reaction following COVID-19 vaccines has been never reported.

A recently published report by Baden et al. [10] describes delayed local-adjacent cutaneous reactions following the administration of the mRNA-1273 vaccine against SARS-CoV-2 in 12 patients enrolled in a phase three trial. The lesions appear after the eight days of receiving the first dose near the injection site after full resolution of the initial local and systemic reactions associated with immunization. All patients received symptomatic treatment, and all symptoms resolved after six days of onset.

The delayed reactions were described as annular, uniformly edematous, targetoid, and/or urticarial plaques, some of which with considerable induration near the vaccination site. A skin biopsy performed on one patient showed superficial perivascular and perifollicular lymphocytic infiltrate with rare eosinophils and scattered mast cells [10].

Nonetheless, neither of these patients developed BCG scar inflammatory responses, most likely because BCG universal vaccination is not part of the US National Immunization Program (NIP).

Moreover, in recent articles, a different, delayed swelling and hypersensitive reaction was noted following a post‐COVID‐19 vaccination and/or flu‐like disease. This reaction was observed in patients who received hyaluronic acid (HA) filler injections as facial “rejuvenation” treatment. It was characterized by the appearance of facial edema and erythema [11-12].

Munavalli et al. [13] mention that even though the mechanism of action for the delayed reaction to HA fillers remains unknown and is likely to be of multifactorial nature, a potential mechanism of delayed inflammatory reactions (DIR) to HA fillers in COVID-19-related cases is the binding and blockade of angiotensin 2 converting enzyme receptors (ACE2), which are targeted by the SARS-CoV-2 virus spike protein to gain entry into the cell. Spike protein interaction with dermal ACE2 receptors favors a pro-inflammatory, loco-regional TH1 cascade, promoting a CD8+T cell-mediated reaction to incipient granulomas, which previously formed around residual HA particles [13].

Nonetheless, we need to remember that a BCG scar is a granuloma induced by an attenuated strain of *Mycobacterium bovis*, in which epithelioid histocytes, fibroblasts, Langerhans´ giant cells, and other type-4 delayed hypersensitivity cells remain there, hence, we do not believe that the pathophysiologic mechanism of a BCG scar is similar to the delayed inflammatory response to HA fillers following a COVID-19 vaccine [14].

We described the “arborizing and comma-shaped vessels” as a unique finding on a BCG scar since these two dermoscopic findings have been associated with other dermatologic conditions such as basal cell carcinoma, dermal nevi, and other, mostly benign conditions [15].

Finally, both the clinical and/or immunological meaning of this reaction related to the medium-long term response to the SARS-CoV-2 vaccines is unknown; hence, based on its frequency, it should be further investigated. However, it is of particular interest that BCG scar changes occur in KD and, most recently in pediatric Multisystem Inflammatory Syndrome associated with COVID-19 (MIS-C); which shares very similar clinical and immunologic findings [16]. And now in our two patients here described associated with COVID-19 vaccine. The immunologic reasons for these striking features are unclear. Only other reports following adult COVID vaccination would help understand these findings and, particularly, when the ongoing clinical trials with pediatric vaccines against SARS-COV-2 are finished and vaccines licensed for children and adolescents.

## Conclusions

These are the first two cases reporting a BCG scar inflammation as a local-adjacent reaction following mRNA COVID-19 vaccine administration. The immunological and clinical implication of this reaction needs to be further assessed. Both clinicians and vaccinators need to be aware to identify and address this secondary local-adjacent reaction to any COVID-19 vaccines, particularly in countries where BCG is part of their NIP since this reaction is likely to cause concerns among patients and requests for evaluation and pharmacovigilance activities. Lastly, it may also encourage vaccine uptake hesitancy.
